# Impact of Naturally Contaminated Substrates on *Alphitobius diaperinus* and *Hermetia illucens*: Uptake and Excretion of Mycotoxins

**DOI:** 10.3390/toxins11080476

**Published:** 2019-08-18

**Authors:** Giulia Leni, Martina Cirlini, Johan Jacobs, Stefaan Depraetere, Natasja Gianotten, Stefano Sforza, Chiara Dall’Asta

**Affiliations:** 1 Department of Food and Drug, University of Parma, Parco Area delle Scienze 27/A, 43124 Parma, Italy; 2 Circular Organics, Slachthuisstraat 120/6, 2300 Turnhout, Belgium; 3 Protifarm, Harderwijkerweg 141B, 3852 AB Ermelo, The Netherlands

**Keywords:** *Alphitobius diaperinus*, *Hermetia illucens*, edible insects, mycotoxin, uptake, excretion, feed safety

## Abstract

Insects are considered a suitable alternative feed for livestock production and their use is nowadays regulated in the European Union by the European Commission Regulation No. 893/2017. Insects have the ability to grow on a different spectrum of substrates, which could be naturally contaminated by mycotoxins. In the present work, the mycotoxin uptake and/or excretion in two different insect species, *Alphitobius diaperinus* (Lesser Mealworm, LM) and *Hermetia illucens* (Black Soldier Fly, BSF), grown on naturally contaminated substrates, was evaluated. Among all the substrates of growth tested, the *Fusarium* toxins deoxynivalenol (DON), fumonisin 1 and 2 (FB1 and FB2) and zearalenone (ZEN) were found in those based on wheat and/or corn. No mycotoxins were detected in BSF larvae, while quantifiable amount of DON and FB1 were found in LM larvae, although in lower concentration than those detected in the growing substrates and in the residual fractions. Mass balance calculations indicated that BSF and LM metabolized mycotoxins in forms not yet known, accumulating them in their body or excreting in the faeces. Further studies are required in this direction due to the future employment of insects as feedstuff.

## 1. Introduction

Given the large growth of the World population expected in the coming years, it has been estimated that the demand for food will raise of about the 60% in 2050 [1]. Along the food chain, the meat production represents the field with the most impact, with serious consequences on the demand of feed supply. Edible insects have been explored as an alternative to common livestock, and their use is encouraged for their sustainability and the minimal environmental impact applied in their breeding [2,3]. Furthermore, edible insects have been proposed as a promising alternative nutrient source due to the high content and quality of their macronutrients [4]. In general, they have a well-balanced nutrient profile, high in polyunsaturated fatty acids and essential amino acids which meet the requirement for humans and livestock, rich in micronutrients and vitamins [5].

In the European Union, the use of insects in the feed and food sector is nowadays regulated by a package of legislative texts. Insects are included in the category of Novel Foods and the European Food Safety Authority (EFSA) authorization is mandatory before their marketing in the European Union (EU) [6]. As alternative source for feedstock, they may substitute the sources of protein and fat normally added in animal feed, as soy, maize, grain and fishmeal [7]. Insects can be used as whole or processed. Whole insects, alive or dried, and fat fraction can be employed for livestock feed, except for aquaculture [8]. Whereas, the protein fraction isolated from insects can be used to feed pet and fur animals, but not for ruminants and monogastrics [9]. Recently, the European Commission (EC) has expanded also to the aquaculture sector, the addition of processed proteins from seven insect species: Black Soldier Fly (*Hermetia illucens*), Common Housefly (*Musca domestica*), Yellow Mealworm (*Tenebrio molitor*), Lesser Mealworm (*Alphitobius diaperinus*), House Cricket (*Acheta domesticus*), Banded Cricket (*Gryllodes sigillatus*) and Field Cricket (*Gryllus assimilis*) [8].

One of the limitations in the use of insects as feed and food is certainly linked to safety aspects. The potential hazards are related to exogenous and endogenous factors, which could be influenced also by harvesting and processing methods, and which could be achieved during all the insect life-cycle [4,10]. Exogenous factors may occur from the behavior and from the substrate of growth which, as feed, must meet the requirements of the current regulation that fixed the maximum limits of undesirable substances in feedstock [11,12]. In particular, the potential hazards could be heavy metal residues, pesticides and mycotoxins. 

Mycotoxins are a wide range of different substances, produced by the secondary metabolism of various species of fungi, that can infect cereal or vegetable crops at pre- or post-harvest [13]. Mycotoxins are characterized by a large chemical diversity and may exert a broad spectrum of adverse effects in animals and humans [14,15]. In addition, they can undergo biotransformation in plants, microbes and animals [16] leading to the uptake of modified forms which may account for a similar toxicity compared to parent compounds [14].

Although mycotoxin occurrence in food and feed is extensively covered by regulation/guidelines at EU level, their presence at concentration levels not exceeding the EU limits cannot be ruled out in rearing substrates obtained from vegetable waste. Therefore, insects are potentially exposed to mycotoxins when reared on a contaminated substrate and, at least from a theoretical point of view, they could accumulate mycotoxins (or modified forms) at levels exceeding the legal limits. 

However, despite a growing interest, only few papers have addressed the possible uptake and biotransformation of mycotoxins in insects so far [17,18,19,20,21,22]. Although information on the possible biotransformation and/or excretion mechanisms are still scattered, all the studies performed to date consistently demonstrated that parent mycotoxins are not bioaccumulated by insects grown in contaminated substrates. 

However, many of these studies were done on substrates artificially contaminated with mycotoxins, possibly not exactly describing the situation arising in the case of a natural contamination. 

The aim of this research, in the framework of the EU project InDIRECT, was to investigate the possible uptake and excretion of mycotoxins in two species of insect larvae, Lesser Mealworm (LM) and Black Soldier Fly (BSF), produced on different naturally contaminated substrates, obtained from vegetable and cereals waste. 

## 2. Results 

Different feed materials were selected for the experiments, in order to ensure optimal growth of the larvae on the basis of preliminary trials. These wastes derived from the processing of cereals, as wheat, corn, rice, while others were chosen among vegetables, as olive and apple pomace, rapeseed and chopped carrots (Table 1).

Samples were analysed for all the regulated mycotoxins, according to the possible occurrence. In particular, samples were analysed for *Fusarium* toxins (deoxynivalenol (DON), fumonisins 1 and 2 (FB1 and FB2), zearalenone (ZEN)) as well as aflatoxins, while the possible presence of patulin was checked in vegetable-based samples. All samples underwent ochratoxin (OTA) analysis, in consideration of its possible synthetisation postharvest in all the considered feed materials. As shown in Table 1, only *Fusarium* toxins were found in three out of eight matrices, at concentration levels in agreement with the EU limits for cereal-based feed. While only DON (938 ± 100 µg/kg) was found in wheat middlings, the contemporary presence of DON, FB1, FB2 and ZEN was detected in corn wastes. In order to optimize and promote the insect growth, 15 feed formulations were obtained by mixing these substrates (originated from the same batch) in different percentages. In particular, corn distillation residues were mixed with chopped carrots, olive and apple pomace, while wheat middlings with corn gluten feed, rice bran and rapeseed, as indicated in Table 2.

According to the contamination pattern observed in raw waste materials, BSF and LM larvae were analysed for the target mycotoxins and results are reported in Table 2. No mycotoxin uptake was observed in BSF larvae, while DON was detected in six out of 13 LM larvae samples, being one contaminated by FB1 as well.

In particular, DON was detected in samples of LM larvae grown on substrates prepared with high percentages of wheat and/or corn residues, and the amount of contamination ranged between 416 ± 28 µg/kg of larvae produced on 100% of wheat wastes and 755 ± 134 µg/kg of insects cultivated on wheat (90%) added with rice (10%). Low concentrations of FB1 (127 ± 6 µg/kg) were detected in LM larvae grown on a substrate composed of 100% of corn gluten feed. 

In order to evaluate the uptake and possible excretion of mycotoxins, the residual fractions were also analysed. All the results are listed in Table 3. Interestingly, the contemporary presence of DON, FB1, FB2 and ZEN was observed in the residual fraction obtained from BSF larvae grown on 100% corn residues, while DON and FB1 were detected in residual fractions from LM corn-based growing substrates. No residual contamination was observed in wheat-based residual fractions, in spite of the DON occurrence detected in wheat waste.

**Figure 1:**
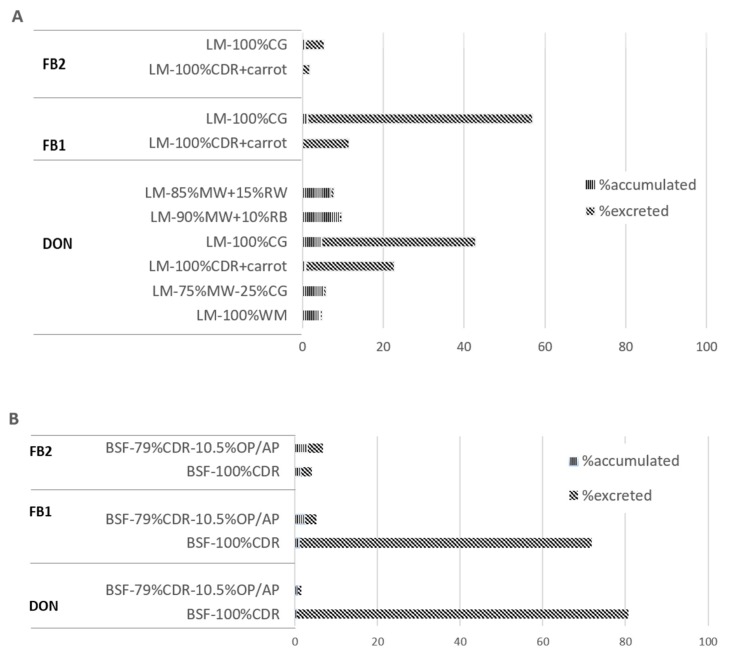
Mass balance of deoxynivalenol (DON), fumonisins 1 and 2 (FB1 and FB2) in Lesser Mealworm (*Alphitobius diaperinus*, LM) (**A**) and Black Soldier Fly (*Hermetia illucens*, BSF) (**B**) treatments. Abbreviations: corn distillation residues, CDR; olive pomace, OP; apple pomace, AP; wheat middlings, WM; corn gluten feed, CG; rice bran, RB; rapeseed wastes, RW.

In order to better investigate the amount of mycotoxins and its distribution in the larvae and in the residual fraction, the mass balance was calculated for larvae which had been resulted positive to the presence of mycotoxins, or grown on contaminated substrates, as in the case of BSF. This calculation was performed on the basis of the concentration of contaminants found in feed, in larvae and in the respective residual fractions. The samples for which the amount of mycotoxins resulted below the LOD were considered as equal to LOD values. Results are represented in Figure 1. 

As far as DON concerned, the mass balance ranged from 2 to 81% in BSF, while it was found to be between 1% and 43% in LM. The average mass balance of fumonisins in BSF ranged between 4% and 72%, and it was ranged from 1% to 57% in LM for FB2 and FB1 respectively. Almost the total amount of ZEN resulted excreted in BSF (data not shown in figure).

## 3. Discussion

This study is focused on the uptake of mycotoxins in BSF and LM larvae, grown on naturally contaminated substrates, obtained from residues of cereal and vegetable processing.

In particular, BSF were reared on two different substrates based on corn distillation residues, which had been found to be contaminated by DON, FB1 and FB2. In agreement with other studies from the literature [21], no uptake of mycotoxins in BSF larvae was observed for both trials, while DON, FB1 and FB2 were found only in the residual fractions collected from the BSF reared on 100% of pure substrate. In this case, the concentration of DON, FB1 and FB2 found in the rests were higher as compared to the concentrations detected in feed. Interestingly, ZEN, lower than LOD in the initial feed, was detected in the residual fraction at 334 ± 44 µg/kg. Since the insects were exclusively grown on the selected substrate, the occurrence of ZEN after harvesting suggested its possible cleavage from the matrix, due to a hydrolytic activity carried out by the insect. An overall increase of ZEN in BSF larvae growing substrates was already observed by Camenzuli et al. [20], although the authors measured the parent compound together with its major phase I metabolites, α- and β-ZEL.

It is well-known that *Fusarium* mycotoxins can be biotransformed by plants into phase I and phase II metabolites, being the glycosylation the most common pathway [16]. Conjugates mycotoxins can be however cleaved by microbial enzymes, as reported by several authors [16,23,24]. Although a hydrolytic activity in insects towards modified mycotoxins has never been described so far, it cannot be ruled out and can be a possible explanation for the observed data.

In addition, it is known that *Fusarium* mycotoxins are often associated to the matrix, and this binding strongly affect the extractability [25,26]. Therefore, taking into consideration the increase of mycotoxins from starting materials to post-growing residues, it can be argued that BSF larvae may induce the substrate degradation upon growing, thus increasing the overall extractability of mycotoxins.

In contrast with BSF larvae, DON was found in LM larvae grown on contaminated substrates, mainly in those containing wheat middlings in combination with corn gluten. When 100% corn gluten was used as growing substrate, DON, FB1 but not ZEN were transferred to LM larvae.

The lack of ZEN uptake in Lesser Mealworm is in agreement with the literature [19], while the possible transferal of DON and FB1 in LM larvae grown on naturally incurred substrates, was observed in this study for the first time.

In case of LM residual fractions, the overall mass balance never exceeded 60%, clearly indicating that mycotoxins are partially metabolised by the larvae to unknown compounds, in agreement with the literature [19,20]. It should be noticed that the occurrence of DON in residual fractions was lower than the one reported in previous studies performed on Yellow mealworm larvae under similar conditions [18]. However, uptake, biotransformation and excretion of mycotoxins in insects could be affected by a range of factors, among them the substrate, the species, and the dose, as well as the use of naturally incurred or spiked growing material [20].

Different from other studies, in which larvae were grown on a substrate artificially contaminated by mycotoxins, at higher concentrations than those found in the substrate samples considered in this study [18,19,20], the present work clearly demonstrated that DON and FB1 can be found in LM (but not in BSF) larvae. The amount of mycotoxins anyway never exceeds the starting levels, indicating that there is no active uptake in insects, and there is rather a degradation or an excretion.

In addition, the mass balance calculation clearly indicated that biotransformation is rather the operating mechanism instead of simple excretion. Further experiments will be needed in order to investigate the mycotoxin biotransformation pattern in insects.

## 4. Conclusions

The present study reported on the possible uptake and/or excretion of mycotoxins in two insect species, LM and BSF, reared on naturally contaminated substrates. As feed, organic side streams recovered from cereal and vegetable processing were considered under a circular economy perspective.

Collected data clearly indicated that transfer from the waste to the insect of mycotoxins is possible, but without uptake into insects, and rather with an overall decrease of their amount. LM larvae were found able to transfer DON and FB in low amounts from naturally incurred growing substrates. Data were consistent with the possible biotransformation of mycotoxins in unknown metabolites in insects. ZEN was detected in BSF residual fractions but not in starting materials, suggesting a possible hydrolytic activity carried out by larvae upon growing.

Taken all together, our results proved the urgency of better deciphering the ability of insects to uptake, transform, and excrete mycotoxins, in view of a safer use of insects as an alternative protein source.

## 5. Materials and Methods

### 5.1. Chemicals

Mycotoxin standard solutions of aflatoxins B1, B2, G1 and G2, fumonisin B1 and B2, A and B trichothecenes (nivalenol, deoxynivalenol, 3-acetyl-deoxynivalenol, fusarenone X, diacetoxyscirpenol, T-2 toxin, HT-2 toxin), zearalenone, ochratoxin A, and patulin were obtained from Romer Labs (Tulln, Austria). All the solvents applied for both the extraction and analysis steps, methanol, acetonitrile formic acid and acetic acid, were HPLC-grade and were purchased from Sigma-Aldrich (Milan, Italy), while Bi-distilled water was produced in-house by using a Milli-Q System (Millipore, Bedford, MA, USA). Salts used for extraction as for the preparation of the eluents as sodium chloride and ammonium acetate were obtained from Sigma-Aldrich (Milan, Italy).

### 5.2. Insect Treatments and Sampling

For the experiments described herein, two insect species were selected: *Alphitobius diaperinus* (Lesser Mealworm, LM) and *Hermetia illucens* (Black Soldier Fly, BSF). The larvae were grown on different substrates prepared using different feed materials, prevalently coming from the processing of cereals (wheat, corn, rice and rapeseed) and vegetable (apple and olive), as indicated in Table 1. The choice of these substrates was driven by the seasonality, the availability and the cost of different by-products of agriculture sector. The substrates were analysed previously for mycotoxins presence, then also larvae were extracted and analysed using the same protocol applied for feed samples. Moreover, rests of insects grown on substrates resulted positive to the presence of mycotoxins were also collected and analysed.

Insects were grown on naturally contaminated feed, and in particular 2 substrates were used for BSF cultivation, while 13 different feeds were prepared for LM production (Table 2). The insects rearing was conducted as indicated in a previous work by Leni et al. [27]. Briefly, BSF eggs were initially placed in a specific incubator at 28 °C for 2 days, then the eggs were transferred in the rearing bins and the new-born larvae treated for 2 days with a started feed composed of chicken feed, the feed materials selected for the experiment and water, with a total dry matter of about 30%. During this time the temperature was set at 28–32 °C and humidity at 60% minimum. After that, the growth substrate was removed and substituted with that selected for the experiment, provided ad libitum. The larvae were reared under these conditions for 15 days. After this period, larvae were removed and quantified, and samples of remaining fractions were also collected as listed in Table 3. Similarly, LM larvae were reared utilizing the selected feed materials chosen for the experiments, under the same controlled conditions of temperature and humidity used for BSF growth, providing feed daily ad libitum. In this case, larvae were harvested for 28 days and then collected. As for BSF, also samples of remaining fractions were recovered and weighted.

Larvae of BSF and LM were killed at −18 °C and stored at the same temperature before each analysis. At the same time, samples of growth substrates and remaining fraction were stored at −18 °C until analyses.

### 5.3. Mycotoxins Extraction and Purification

Mycotoxins class is represented by several organic compounds with different chemical and physical properties. For this reason, we decided to apply different extraction protocols, selected for a specific class of toxins. In addition, all the samples of insect larvae and samples of remaining fractions were subjected to a lyophilisation process (Freeze dryer Lio-5P, 5Pascal, Milano, Italy) for 48 h and milled using a laboratory miller. The dried powders obtained from these steps were stored at −20 °C until extraction and analysis.

For the extraction of aflatoxins, 1g of sample added with 0.2 g of NaCl was extracted using 4 mL of a mixture of methanol/bi-distilled water, 80/20 *v*/*v* on a shaker at room temperature, at 200 strokes/min for 90 min. After that, the extract was centrifuged at 10,621× *g*, at 25 °C for 10 min. 1 mL of the supernatant was transferred in a tube, diluted with 4 mL of bi-distilled water and submitted to a purification step using immuno-affinity columns (VICAM, Afla Test^®^, mycotoxin testing system; VICAM, Milford, MA, USA). The cartridges were conditioned with 10 mL of bi-distilled water and subsequently with 10 mL of pure methanol. The diluted extract was then eluted through the column and, after the elution, a washing step with 10 mL of bi-distilled water was performed. The analytes were recovered with 1 mL of pure methanol. The purified sample was dried under a gently nitrogen flow, suspended in 1 mL of bi-distilled water/methanol, 80/20 *v*/*v*, and analysed by HPLC-FLD technique.

The contemporary extraction of fumonisins, ochratoxin A, zearalenone, patulin and A and B trichothecenes was performed on the basis of different protocols with slight modifications [28,29]. Briefly, 1 g of sample was extracted adding 4 mL of a solution composed of bi-distilled water/acetonitrile/methanol 50/25/25 *v*/*v*. The sample was positioned on a shaker at room temperature, at 200 strokes/min for 90 min. After that, the extracts were centrifuged at 10,621× *g*, for 10 min at 25 °C. 1 mL of the supernatant was collected and dried under a gently nitrogen flow. The residue was then dissolved in 1 mL of bi-distilled water/methanol 80/20 *v*/*v*. The samples were then subjected to UHPLC-MS/MS analyses.

### 5.4. Mycotoxins Analysis

Aflatoxins B1, B2, G1 and G2 were analysed on a HPLC Waters Alliance 2695 separation module, coupled with a FLD detector (Waters, Multi λ Fluorescence detector 2475) and an UV detector (Waters, Dual λ Absorbance Detector 2489) (Waters, Milford, MA, USA). The analytes separation was achieved on a C18-RP XTerra (Waters, Milford, MA, USA; 250 × 2.1 mm, i.d. 5 mm) column using as eluents bi-distilled water (A) and methanol (B), in isocratic conditions (65% A and 35% B). The flow was set at 0.25 mL/min and the column oven temperature was kept at 30 °C. A volume of 10 µL was injected. For the detection of aflatoxins, the UV detector was set at λ = 365 nm, while for the FLD λ = 365 nm and λ = 425 nm were chosen as the typical wavelength of absorbance and of emission, respectively.

For the quantitative determination of aflatoxins, a calibration curve was prepared starting from the commercial standard which contained AFB1 and AFG1 at the concentration of 2 mg/kg and AFB2 and AFG2 at the concentration of 0.5 mg/kg. Starting from this solution, 5 different dilutions in pure methanol were performed obtaining AFB1 and AFG1 at 0.5, 0.75, 1, 1.5 and 2 µg/kg, while for AFB2 and AFG2 concentrations of 0.125, 0.187, 0.25, 0.375 and 0.5 µg/kg, obtaining a good linearity (R^2^ > 0.99) for the both calibration ranges.

Fumonisins B1 and B2, ochratoxin A, zearalenone, patulin and A and B trichothecenes (nivalenol, deoxynivalenol, 3-acetyl-deoxinivalenol, fusarenone X, T2 toxin, HT2 toxin and deacetoxyscirpenol) were determined on an UHPLC–MS/MS apparatus consisted of an UHPLC Ultimate 3000 separation module (Dionex, Sunnyvale, CA, USA), coupled with a TSQ Vantage triple quadrupole (Thermo Fisher, Waltham, MA, USA) equipped with an ESI interface. The separation of the analytes was achieved on a RP-C18 EVO Kinetex column (2.6 μ, 100A; 100 × 2.10 mm) from Phenomenex (Torrance, CA, USA). Ammonium acetate 5 mM in bi-distilled water and methanol were used as eluent A and B respectively, both acidified with the 0.2% of acetic acid. A gradient was applied as follows: the elution started with 2% of B and these conditions were maintained for 1 min, then at 2 min the percentage of B was increased at 20% and kept for 6 min, at 17 min the column was flashed with the 90% of B for 3 min, then in 1 min the initial conditions were re-established and the column was re-equilibrated for 9 min, with a total run time of 30 min. During the analyses the column temperature was maintained at 40 °C while samples were maintained at 20 °C. The flow was 0.35 mL/min and for each sample 4 µL were injected into the system.

PAT, NIV, DON, 3ADON, FUSX, and ZEN were monitored in negative ion mode with a spray voltage of 3500 V, a capillary temperature of 270 °C, a vaporizer temperature of 200 °C, a sheath gas flow of 50 units and an auxiliary gas flow of 5 units. T2, HT2 toxins, DAS, FB1, FB2, and OTA were monitored applying a positive ionization mode, with the following parameters: spray voltage of 3000 V, a capillary temperature of 270 °C, a vaporizer temperature of 200 °C, a sheath gas flow of 50 units and an auxiliary gas flow of 5 units. All the other parameters as S-Lens RF amplitude values were obtained and set by tuning methanolic solutions of each considered molecule (1 mg/kg).

Detection of all the considered analytes was performed in SRM modality (Single Reaction Monitoring) monitoring the characteristic transitions for each considered mycotoxin (Table 4).

In order to quantify these mycotoxins, a calibration curve containing all the considered analytes was prepared starting from the commercial standard. For this purpose, 6 different dilutions were prepared considering the following concentrations: 50, 100, 200, 500, 750 and 1000 µg/kg, obtaining a good linearity (R^2^ > 0.99) for the calibration range.

### 5.5. Mass Balance Calculation

The mass balance was calculated as described by Camenzuli et al. [20] on the basis of the amount of substrates used for insects’ growth, the amount of harvested larvae and of the residual fractions (frass). Furthermore, the accumulated, extracted and potential metabolized mycotoxins were calculated as follow:

$$\%\;\mathrm{accumulated}\;\mathrm{mycotoxin}=\frac{\;\mathrm{amount}\;\mathrm{of}\;\mathrm{harvested}\;\mathrm{insects}\;x\;\mathrm{concentration}\;\mathrm{mycotoxin}\;\mathrm{detected}\;\mathrm{in}\;\mathrm{insects}}{\mathrm{amount}\;\mathrm{of}\;\mathrm{substrates}\;x\;\mathrm{concentration}\;\mathrm{mycotoxin}\;\mathrm{detected}\;\mathrm{in}\;\mathrm{substrates}}\;\times \;100,$$

$$\%\;\mathrm{excreted}\;\mathrm{mycotoxin}=\frac{\;\mathrm{amount}\;\mathrm{of}\;\mathrm{frass}\;x\;\mathrm{concentration}\;\mathrm{mycotoxin}\;\mathrm{detected}\;\mathrm{in}\;\mathrm{frass}}{\mathrm{amount}\;\mathrm{of}\;\mathrm{substrates}\;x\;\mathrm{concentration}\;\mathrm{mycotoxin}\;\mathrm{detected}\;\mathrm{in}\;\mathrm{substrates}}\;\times \;100,$$

% metabolyzed mycotoxin= 100− % excreted mycotoxin− % accumulated mycotoxin.

Metabolized mycotoxins were referred to the undetected compounds which could be metabolized in different structures not yet identified. All the measurements of mycotoxin below the LOD were considered as equal to LOD values.

## Figures and Tables

**Table 1:** Substrates used for feed formulations with results about their mycotoxin contamination (deoxynivalenol (DON), fumonisins 1 and 2 (FB1 and FB2), zearalenone (ZEN)). Results showed the detected mycotoxins and are reported as mean of two different replicates ± standard deviation.

Substrate Samples		Mycotoxin Amount (µg/kg)			
Sample Code	Description	DON	FB1	FB2	ZEN
WM	Wheat middlings	938 ± 100	<LOD	<LOD	<LOD
CDR	Corn distillation residues	779 ± 5	573 ± 3	441 ± 3	<LOD
CG	Corn gluten feed	1207 ± 43	727 ± 6	294 ± 5	173 ± 4
RB	Rice Bran	<LOD	<LOD	<LOD	<LOD
RW	Rapeseed wastes	<LOD	<LOD	<LOD	<LOD
OP	Olive pomace	LOD	<LOD	<LOD	<LOD
AP	Apple pomace	<LOD	<LOD	<LOD	<LOD
CC	Chopped carrots	<LOD	<LOD	<LOD	<LOD

**Table 2:** Larvae of Black Soldier Fly (*Hermetia illucens*, BSF) and Lesser Mealworm (*Alphitobius diaperinus*, LM) reared on the feed formulations with results about the target mycotoxins occurrence. Results are the mean of two different replicates and are reported as mean ± standard deviation. Abbreviations: deoxynivalenol, DON; fumonisins 1 and 2, FB1 and FB2; zearalenone, ZEN; corn distillation residues, CDR; olive pomace, OP; apple pomace, AP; wheat middlings, WM; corn gluten feed, CG; rice bran, RB; rapeseed wastes, RW.

Larvae Samples		Mycotoxin Amount (µg/kg)			
Sample Code	Description	DON	FB1	FB2	ZEN
BSF-100% CDR	BSF larvae grown on: 100% CDR	<LOD	<LOD	<LOD	<LOD
BSF-79% CDR-10.5% OP/AP	BSF larvae grown on: 79% CDR, 10.5% OP, 10.5% AP	<LOD	<LOD	<LOD	<LOD
LM-100% WM	LM larvae grown on: 100% WM, 0% CG	416 ± 28	<LOD	<LOD	<LOD
LM-75% WM-25% CG	LM larvae grown on: 75% WM, 25% CG	608 ± 59	<LOD	<LOD	<LOD
LM-50% WM-50% CG	LM larvae grown on: 50% WM, 50% CG	<LOD	<LOD	<LOD	<LOD
LM-100% CG	LM larvae grown on: 100% CG	726 ± 164	127 ± 6	<LOD	<LOD
LM-100% CDR *	LM larvae grown on: 100% CDR *	468 ± 181	<LOD	<LOD	<LOD
LM-95% WM-5% RB	LM larvae grown on: 95% WM, 5% RB	<LOD	<LOD	<LOD	<LOD
LM-90% WM-10% RB	LM larvae grown on: 90% WM, 10% RB	755 ± 134	<LOD	<LOD	<LOD
LM-85% WM-15% RB	LM larvae grown on: 85% WM, 15% RB	<LOD	<LOD	<LOD	<LOD
LM-80% WM-20% RB	LM larvae grown on: 80% WM, 20% RB	<LOD	<LOD	<LOD	<LOD
LM-95% WM-5% RW	LM larvae grown on: 95% WM, 5% RW	<LOD	<LOD	<LOD	<LOD
LM-90% WM-10% RW	LM larvae grown on: 90% WM, 10% RW	<LOD	<LOD	<LOD	<LOD
LM-85% WM-15% RW	LM larvae grown on: 85% WM, 15% RW	557 ± 237	<LOD	<LOD	<LOD
LM-80% WM-20% RW	LM larvae grown on: 80% WM, 20% RW	<LOD	<LOD	<LOD	<LOD

* A small amount of chopped carrots was arbitrarily added in order to get the desired water content for optimal insect growth.

**Table 3:** Residual fractions harvested from insects resulted positive to the presence of target mycotoxins and results about their concentration level expresses as µg/kg. Results are the mean of two different replicates and are reported as mean ± standard deviation. Abbreviations: black soldier fly, BSF; lesser mealworm, LM; deoxynivalenol, DON; fumonisins 1 and 2, FB1 and FB2; zearalenone, ZEN; corn distillation residues, CDR; olive pomace, OP; apple pomace, AP; wheat middlings, WM; corn gluten feed, CG; chopped carrots, CC; rice bran, RB; rapeseed wastes, RW.

Residual Fraction Samples		Mycotoxin Amount (µg/kg)			
Sample Code	Description	DON	FB1	FB2	ZEN
REST-BSF-100% CDR	Rests of BSF larvae grown on: 100% CDR	1473 ± 197	951 ± 152	344 ± 64	334 ± 44
REST-BSF-79% CDR-10.5% OP/AP	Rests of BSF larvae grown on: 79% CDR, 10.5% OP, 10.5% AP	<LOD	<LOD	<LOD	<LOD
REST-LM-100% WM	Rests of LM larvae grown on: 100% WM, 0% CG	<LOD	<LOD	<LOD	<LOD
REST-LM-75% WM-25% CG	LM larvae grown on: 75% WM, 25% CG	<LOD	<LOD	<LOD	<LOD
REST-LM-100% CG	Rests of LM larvae grown on: 100% CG	827 ± 61	728 ± 7	<LOD	<LOD
REST-LM-100% CDR *	Rests of LM larvae grown on: 100% CDR *	587 ± 73	224 ± 8	<LOD	<LOD
REST-LM-90% WM-10% RB	Rests of LM larvae grown on: 90% WM, 10% RB	<LOD	<LOD	<LOD	<LOD
REST-LM-85% WM-15% RW	Rests of LM larvae grown on: 85% WM, 15% RW	<LOD	<LOD	<LOD	<LOD

* A small amount of chopped carrots was arbitrarily added in order to get the desired water content for optimal insect growth.

**Table 4:** Characteristic transitions monitored for the target mycotoxins: fumonisins B1 and B2 (FB1, FB2), ochratoxin A (OTA), zearalenone (ZEN), patulin (PAT) and A and B trichothecenes (nivalenol (NIV), deoxynivalenol (DON), 3-acetyl-deoxinivalenol (3ADON), fusarnone X (FUSX), T2 toxin, HT2 toxin and deacetoxyscirpenol (DAS)).

Compound	Ionization Mode	Precursor Ion (*m*/*z*)	Product Ions (*m*/*z*)	Collision Energy (V)	LOD (µg/kg)
PAT	Negative	152.9 [M − H]^−^	109/81	−12/−12	100
NIV	Negative	371.1 [M + CH3COO]^−^	311.1/281.1/59.1	−10/−32/−48	10
DON	Negative	355.1 [M + CH3COO]^−^	295.1/265.1	−13/−16	10
3ADON	Negative	397.1 [M + CH3COO]^−^	307.1/59	−18/−20	20
FUSX	Negative	413.3 [M + CH3COO]^−^	353.6/262.9/59.1	−14/−22/−10	20
OTA	Positive	404.5 [M + H]^+^	238.7/220.7/101.7	21/31/68	20
FB1	Positive	722.3 [M + H]^+^	704.7/352.1/334.1	26/35/38	25
FB2	Positive	706.5 [M + H]^+^	688.4/336.3	51/51	25
DAS	Positive	384.2 [M + NH_4_]^+^	307.2/105.1	17/61	10
T2	Positive	484.3 [M + NH_4_]^+^	215.0/185.0	19/22	10
HT2	Positive	442.0 [M + NH_4_]^+^	263.1	11	10
ZEN	Negative	317.0 [M − H]^−^	175.0/131.0	−26/−32	10
